# Expanding the scope of candidate prognostic marker IGFBP2 in glioblastoma

**DOI:** 10.1042/BSR20190770

**Published:** 2019-07-19

**Authors:** Mikael S. Lindström

**Affiliations:** Division of Genome Biology, Department of Medical Biochemistry and Biophysics, Karolinska Institutet, Stockholm SE-171 21, Sweden

**Keywords:** biomarkers, glioma, insulin-like growth factor

## Abstract

Glioblastoma is the most common malignant brain tumor in adults. Unfortunately, it has a very poor prognosis and no cure. In a recent paper by Yuan et al. (*Bioscience Reports* (2019), DOI:10.1042/BSR20190045) RNAscope was used to detect insulin-like growth factor binding protein 2 (*IGFBP2*) mRNA in glioblastoma biopsies. The study revealed that patients with high levels of *IGFBP2* mRNA had shorter survival and that *IGFBP2* transcript level was an independent prognostic factor. It is also of value to determine the prognostic effect of *IGFBP2* on established biomarkers such as isocitrate dehydrogenase (*IDH1*) mutations or telomerase reverse transcriptase (*TERT*) promoter mutation. In the present study, the combination of having a *TERT* promoter mutation, and at the same time a high level of *IGFBP2* mRNA, was associated with very poor survival rates. It was concluded that *IGFBP2* predicts the survival of the patients with *TERT* promoter mutation. This finding may have important implications for glioblastoma prognosis. IGFBP2 re-emerges as a candidate biomarker and potential therapeutic target in glioma. Further research into its functional roles during glioma progression may provide additional insights into this deadly disease.

Glioblastoma multiforme (GBM) corresponding to World Health Organization (WHO) grade IV glioma is the most deadly brain tumor in adults with a median overall survival of approximately 15 months from diagnosis [1]. Despite intensive treatment including surgical resection, radiotherapy, and chemotherapy the average 5-year survival rate is less than 10% [1,2]. Introduction of temozolomide in 2005 resulted in a modest increase in survival, mostly restricted to younger patients [3]. One of the many challenges with glioblastomas is the infiltrative and migrating nature of the cancer cells that spread and hide in normal brain regions, a mimic of embryonic glial propagation. Another challenge is the notorious resistance to chemo- and radiotherapy, in part related to the cellular heterogeneity often seen in glioblastoma. Over the past decade, omics-technologies have resulted in a tremendous increase in knowledge of the epigenetic, genetic, and transcriptome alterations found in glioblastomas [4–6]. This has led to definitions of molecularly distinct subtypes, including the gene expression-based subtypes known as proneural, neural, classical, and mesenchymal types. In a rapidly developing field, there are now additional molecular markers and classifications of brain tumors as reflected in the 2016 update of the WHO Classification of Tumors of the Central Nervous System [1]. For example, point mutations in the isocitrate dehydrogenase-1 (*IDH-1*) and *IDH-2* genes classify glioblastoma into IDH-mutant glioblastomas, characterized by epigenetic hypermethylation and proneural gene expression, and IDH-wildtype glioblastomas (neural, proneural, mesenchymal, or classical gene expression patterns) [1,7]. If no mutations are present in *IDH*, the glioblastomas are known as IDH-wildtype (these often correspond to the so-called primary glioblastomas that develop *de novo*). Mutations in the *IDH1* and 2 genes initially occur in WHO grade II and grade III gliomas and are associated with improved survival [1,7]. The majority of glioblastomas can also be divided into molecular subgroups based on mutations in the telomerase reverse transcriptase (*TERT*) promoter [8,9]. These molecular subgroups use different mechanisms to maintain the telomeres, either *TERT* promoter mutation causing telomerase activation or mutations in *ATRX* leading to an alternative lengthening of telomeres [10]. The molecular understanding of gliomas including the glioblastomas is rapidly evolving, and a complete discussion on this topic is outside the scope of this commentary. Regarding the glioblastomas, presence or absence of *IDH* mutation, *MGMT* (O(6)-methyl guanidine-DNA-methyltransferase) promoter methylation, and *TERT* promoter mutations are of particular interest to analyze [9].

Despite intense research and many great discoveries over the past decade, there is still a need to find additional sharp and reliable, easy to analyze biomarkers that can further improve the classification and treatment of brain tumors. Such markers could include diagnostic biomarkers to enable more accurate classification, prognostic biomarkers that inform about a likely cancer outcome and predictive biomarkers to give hints about the best treatment strategy. Today, some glioma-specific molecular biomarkers include *IDH* mutations (grades II, III gliomas), chromosomal region 1p19q deletion, and *MGMT* promoter methylation [9].

Following a previous comparison of gene expression profiles between gliomas with different grades that revealed frequent overexpression of insulin-like growth factor binding protein 2 (*IGFBP2*) in glioblastomas [12], Yuan et al. [11] set out to investigate *IGFPB2* mRNA levels in a larger cohort of glioblastomas. What is known about IGFBP2 in brain tumors? IGFBPs bind and regulate the bioavailability and signaling activity of circulating IGF-I and IGF-II [13]. Often, IGFBP2 expression positively correlates with tumor aggressiveness and other known cancer markers [13]. Functionally, IGFBP2 has been established as a driver of glioma progression to a higher grade [14,15]. IGFBP2-driven tumors are dependent on the continued expression of IGFBP2, as knockdown led to a significant decrease in tumor progression and prolonged survival. Exogenous IGFBP2 increases the proliferation and invasive capacity of the glioma cells, and induces chemoresistance, while knockdown of IGFBP2 resulted in both decreased invasiveness and tumorigenicity in nude mice [14,15]. Evidence suggests that IGFBP2 engages the Akt signaling pathway and at least in some settings collaborates with platelet-derived growth factor β (PDGFB) in the development of glioma [14]. Furthermore, IGFBP2 is overexpressed within the stem cell compartment of glioblastomas and is needed for clonal expansion and proliferation of glioma stem cells, and IGFBP2 may also contribute to tumor progression by enriching for glioma stem cells and boosting their survival [16].

*In situ* analysis of biomarkers is of interest because it allows visualization of the expression pattern within the tumor in relation to other parameters of interest. RNAscope is a novel RNA *in situ* hybridization technology with a unique probe design. This innovative technique may achieve detection of single molecules in individual cells and the assay can be multiplexed if needed. As mentioned, *IGFBP2* overexpression is common in high-grade glioma and *IGFBP2* is a prognostic factor for poor survival [17]. One may wonder why Yuan et al. [11] set out to re-investigate something already known nearly two decades ago [12]. Well, the answer is that studies of *IGFBP2* transcript expression in glioblastoma biopsies detected by an *in situ* method were very few, but as of today, the techniques have been improved and could either confirm or challenge previous results. Innovative technologies could present more robust and reliable data leading to, for example revival and repurposing of ‘forgotten’ biomarkers. In the present study, RNAscope probe was used to detect the expression of *IGFBP2* mRNA in 180 glioblastomas [11]. The analysis revealed 16.9 months median overall survival for the patients with low *IGFBP2* mRNA levels, but only 11.6 months for patients with high *IGFBP2*, which is a striking difference. In other datasets this difference appears less pronounced, but remains significant [17]. Presumably, the resolution of RNAscope allows for a sharper distinction of the expression patterns than immunohistochemistry. It is of further interest to test *IGFBP2* levels and its prognostic value on to already established biomarkers since it may, for example, help to select patients with poor prognosis and who may benefit from more rapid or aggressive therapy. Yuan et al. [11] therefore divided the glioblastoma patients into subgroups. Combining *IGFBP2* mRNA expression and *TERT* promoter status, the survival analysis showed that GBM patients harboring wild-type *TERT* promoter had the longest median overall survival time of 19.6 months. In patients with mutation in the *TERT* promoter, those that had low *IGFBP2* mRNA presented with a median overall survival of 14.8 months, and patients with both mutation in the *TERT* promoter and high levels of *IGFBP2* mRNA had the shortest overall survival of 9.8 months. These are highly distinct differences in survival between the groups.

Recently, IGFBP2 has been put forward as a specific prognostic marker in IDH-mutant low-grade glioma patients [18]. It turns out that IDH-mutant glioma patients generally manifest low *IGFBP2* expression, which is associated with improved survival independent of *IDH* mutational status, whereas high *IGFBP2* expression results in worse survival than in the IDH-wildtype group. Thus for the IDH-mutant group *IGFBP2* is prognostic [18]. In the present study focusing on glioblastomas, Yuan et al. [11] found in agreement a negative correlation between the presence of *IDH* mutation and high level of *IGFPBP2* mRNA. Additional analysis of the data accumulated in relation to *IDH* are certainly of interest.

In summary, *IGFBP2* mRNA served as an independent prognostic biomarker in glioblastomas. It was also revealed that *IGFBP2* mRNA might serve as a potential prognostic indicator together with *TERT* promoter status [11]. The data shown in the present study [11] and in other recent ones [17,18], reveal that IGFBP2 stands out among many other candidate molecular biomarkers. However, biomarkers need to be easy to analyze and provide clear answers in order to improve classification or treatment. With this perspective RNAscope analysis of *IGFBP2* appears demanding, but one may consider it in some settings. Could there be other proxies for *IGFBP2* that are easier to analyze? Of particular interest would be minimally invasive biomarkers for diagnosis and as measures of response to therapeutic interventions, for example those found in serum or that can be detected by imaging. Additional studies are warranted on this topic. Finally, it should be mentioned that IGFBP2 most likely plays an important role in the immunologic processes of glioblastomas including immunosuppressive checkpoints and various signaling pathways [17,19]. Moreover, neutralizing antibodies against IGFBP2 impaired IGFBP2-mediated oncogenic signaling pathways and inhibited the spread of tumor cells [20]. IGFBP2 therefore remains as a candidate biomarker and potential therapeutic target in glioma therapy, perhaps immunotherapy in particular. Further research into its functional roles and regulatory mechanisms during glioma progression may provide additional insights into therapeutic approaches in the management of this deadly disease.

## Abbreviations

IDH: isocitrate dehydrogenase
IGFBP2: insulin-like growth factor binding protein 2
MGMT: O(6)-methyl guanidine-DNA-methyltransferase
TERT: telomerase reverse transcriptase
WHO: World Health Organization

## References

[1] Louis D.N., Perry A., Reifenberger G., von Deimling A., Figarella-Branger D., Cavenee W.K. (2016) The 2016 World Health Organization Classification of Tumors of the Central Nervous System: a summary. Acta Neuropathol. 131, 803–820 10.1007/s00401-016-1545-1 27157931

[2] Stupp R., Mason W.P., van den Bent M.J. (2005) Radiotherapy plus concomitant and adjuvant temozolomide for glioblastoma. N. Engl. J. Med. 352, 987–996 10.1056/NEJMoa043330 15758009

[3] Woehrer A., Bauchet L. and Barnholtz-Sloan J.S. (2014) Glioblastoma survival: has it improved? Evidence from population-based studies Curr. Opin. Neurol. 27, 666–674 25364955 10.1097/WCO.0000000000000144

[4] Parsons D.W., Jones S., Zhang X., Lin J.C.-H., Leary R.J., Angenendt P. (2008) An integrated genomic analysis of human glioblastoma multiforme. Science 321, 1807–1812 10.1126/science.1164382 18772396 PMC2820389

[5] Verhaak R.G.W, Hoadley K.A., Purdom E., Wang V., Qi Y., Wilkerson M.D. (2010) Integrated genomic analysis identifies clinically relevant subtypes of glioblastoma characterized by abnormalities in PDGFRA, IDH1, EGFR, and NF1. Cancer Cell 17, 98–110 10.1016/j.ccr.2009.12.020 20129251 PMC2818769

[6] Cancer Genome Atlas Research Network (2008) Comprehensive genomic characterization defines human glioblastoma genes and core pathways. Nature 455, 1061–1068 10.1038/nature07385 18772890 PMC2671642

[7] Yan H., Parsons D.W., Jin G., McLendon R., Rasheed B.A., Yuan W. (2009) IDH1 and IDH2 mutations in gliomas. N. Engl. J. Med. 360, 765–773 10.1056/NEJMoa0808710 19228619 PMC2820383

[8] Eckel-Passow J.E., Lachance D.H., Molinaro A.M., Walsh K.M., Decker P.A., Sicotte H. (2015) Glioma groups based on 1p/19q, IDH, and TERT promoter mutations in tumors. N. Engl. J. Med. 372, 2499–2508 10.1056/NEJMoa1407279 26061753 PMC4489704

[9] Ruff M.W., Uhm J.H. and Benarroch E.E. (2019) Neuro-oncology: implications of the molecular era. Neurology 92, 568–574 10.1212/WNL.0000000000007126 30760635

[10] Diplas B.H., He X., Brosnan-Cashman J.A., Liu H., Chen L.H., Wang Z. (2018) The genomic landscape of TERT promoter wild-type IDH glioblastoma. Nat. Comm. 9, 2087 10.1038/s41467-018-04448-6 PMC5970234 29802247

[11] Yuan Q., Cai H.Q., Zhong Y., Zhang M.J., Cheng Z.J., Hao J.J. (2019) Overexpression of IGFBP2 mRNA predicts poor survival in patients with glioblastoma. Biosci. Rep. 39, BSR20190045 10.1042/BSR20190045 31138764 PMC6567677

[12] Fuller G.N., Rhee C.H., Hess K.R., Caskey L.S., Wang R., Bruner J.M. (1999) Reactivation of insulin-like growth factor binding protein 2 expression in glioblastoma multiforme: a revelation by parallel gene expression profiling. Cancer Res. 59, 4228–4232 10485462

[13] Baxter R.C. (2014) IGF binding proteins in cancer: mechanistic and clinical insights. Nat. Rev. Cancer 14, 329–341 10.1038/nrc3720 24722429

[14] Dunlap S.M., Celestino J., Wang H., Jiang R., Holland E.C., Fuller G.N. (2007) Insulin-like growth factor binding protein 2 promotes glioma development and progression. Proc. Natl. Acad. Sci. U.S.A. 104, 11736–11741 10.1073/pnas.0703145104 17606927 PMC1913900

[15] Moore L.M., Holmes K.M., Smith S.M., Wu Y., Tchougounova E., Uhrbom L. (2009) IGFBP2 is a candidate biomarker for Ink4a-Arf status and a therapeutic target for high-grade gliomas. Proc. Natl. Acad. Sci. U.S.A. 106, 16675–16679 10.1073/pnas.0900807106 19805356 PMC2757856

[16] Hsieh D. (2010) IGFBP2 promotes glioma tumor stem cell expansion and survival. Biochem. Biophys. Res. Commun. 397, 367–372 10.1016/j.bbrc.2010.05.145 20515648

[17] Cai J., Chen Q., Cui Y., Dong J., Chen M., Wu P. (2018) Immune heterogeneity and clinicopathologic characterization of IGFBP2 in 2447 glioma samples. Oncoimmunology 7, e1426516 10.1080/2162402X.2018.1426516 29721393 PMC5927515

[18] Huang L.E., Cohen A.L., Colman H., Jensen R.L., Fults D.W. and Couldwell W.T. (2017) IGFBP2 expression predicts IDH-mutant glioma patient survival. Oncotarget 8, 191–202 27852048 10.18632/oncotarget.13329 PMC5352106

[19] Preusser M., Lim M., Hafler D.A., Reardon D.A. and Sampson J.H. (2015) Prospects of immune checkpoint modulators in the treatment of glioblastoma. Nat. Rev. Neurol. 11, 504–514 10.1038/nrneurol.2015.139 26260659 PMC4782584

[20] Phillips L.M., Zhou X., Cogdell D.E., Chua C.Y., Huisinga A., R Hess K. (2016) Glioma progression is mediated by an addiction to aberrant IGFBP2 expression and can be blocked using anti-IGFBP2 strategies. J. Pathol. 239, 355–364 10.1002/path.4734 27125842 PMC4915980

